# Supplementary material for: Distinct gene expression and secondary metabolite profiles in suppressor of prosystemin-mediated responses2 (spr2) tomato mutants having impaired mycorrhizal colonization
Source: PeerJ. 2020 Apr 16;8:e8888. doi: 10.7717/peerj.8888 (PMC7167247; doi:10.7717/peerj.8888)
Supplement: Supplemental Information 1 — The mean ± SE of colonization frequency (F%), intensity of mycorrhizal colonization (M%) and arbuscule abundance (A%) in the root system of wild type (WT) and spr2 mutant plants analyzed 50 days post inoculation (dpi). Asterisks over the bars represent statistical difference at P < 0.001 (n = 10). [file peerj-08-8888-s001.docx]

***

***

***

WT

*spr2*

**50 dpi**

Percent colonization

100

75

50

25

0

F% M% A%

**Figure S1. Degree of arbuscular mycorrhizal fungi (AMF) colonization, at 50 dpi, in roots of wild type (WT) and *spr2* tomato plants inoculated with *Rhizophagus irregularis*.** The mean ± SE of colonization frequency (F%), intensity of mycorrhizal colonization (M%) and arbuscule abundance (A%) in the root system of wild type (WT) and *spr2* mutant plants analyzed 50 days post inoculation (dpi). Asterisks over the bars represent statistical difference at *P* ˂ 0.001 (n = 10).
