# Supplementary material for: Distinct gene expression and secondary metabolite profiles in suppressor of prosystemin-mediated responses2 (spr2) tomato mutants having impaired mycorrhizal colonization
Source: PeerJ. 2020 Apr 16;8:e8888. doi: 10.7717/peerj.8888 (PMC7167247; doi:10.7717/peerj.8888)
Supplement: Supplemental Information 2 [file peerj-08-8888-s002.docx]

**Table S1**. Sequence of oligonucleotide primer sequences used for the qPCR analysis.

| *Solyc* number | Gene | **Gene name** |  | 5’ - 3’ sequence |
| --- | --- | --- | --- | --- |
| *Solyc06g051860.1.1* | *LePT4* | *PHOSPHATE TRANSPORTER 4* | Direct | GAAGGGGAGCCATTTAATGTGG |
|  |  |  | Reverse | ATCGCGGCTTGTTTAGCATTTC |
| *Solyc10g086500.1* | *DET 2* | *DE-ETIOLATED 2* | Direct | GTTGGGCCTGAAGAGTCAAG |
|  |  |  | Reverse | AAACAGCTCGAGGAACCAAA |
| *Solyc02g085360.2* | *DWF4* | *DWARF 4* | Direct | GCAGCGCATTTAGATCCTTC |
|  |  |  | Reverse | CCATCTCAAGTTTGGCCAGT |
| *Solyc06g051750.2* | *CPD* | *CONSTITUTIVE PHOTOMORPHOGENESIS AND DWARFISM* | Direct | TGTTTCAAAATACGGCAGCA |
|  |  |  | Reverse | CCGGGATAACTCGACTCAAA |
| *Solyc11g011260.1* | *GAI* | *GIBBERELLIC ACID INSENSITIVE* | Direct | TGGGTCTTCGTCTTCAGCTT |
|  |  |  | Reverse | TTGAACCCAGATGAACCG |
| *Solyc07g056570.1* | *NCED1* | *9-CIS-EPOXYCAROTENOID DIOXYGENASE 1* | Direct | ACCCACGAGTCCAGATTTC |
|  |  |  | Reverse | GGTTCAAAAAGAGGGTTAGC |
| *Solyc02g084850.2* | *TAS14* | *ABSCISIC ACID AND ENVIRONMENTAL STRESS-INDUCIBLE PROTEIN 14* | Direct | ACTCAAGGCATGGGTACTGG |
|  |  |  | Reverse | CCTTCTTTCTCCTCCCACCT |
| *Solyc07g049690.2* | *HPL* | *HYDROPEROXIDE LYASE* | Direct | TCTCTTCTTCCTGCACTCCA |
|  |  |  | Reverse | CCAATGCTGACAGTAGGTGC |
| *Solyc08g014000.2* | *LOX A* | *LIPOXYGENASE A* | Direct | GGTTACCTCCCAAATCGTCC |
|  |  |  | Reverse | TGTTTGTAACTGCGCTGTG |
| *Solyc10g007960.1* | *AOS3* | *ALLENE OXIDE SYNTHASE 3* | Direct | GCGGAGGAGTTCAATCCAG |
|  |  |  | Reverse | CGCATGAAAAACTCCACAACC |
| *Solyc01g109140.2* | *DES* | *DIVINYL ETHER SYNTHASE* | Direct | CCGGATGAGTTTGTACCTGA |
|  |  |  | Reverse | ATCTTTGCCTGGACATTGCT |
| *Solyc03g122340.2* | *LOX D* | *LIPOXYGENASE D* | Direct | GGTGTCATCGAGGCTTGC |
|  |  |  | Reverse | CCCTGTCACTGCAAACTTG |
| *Solyc04g079730.1* | *AOS1* | *ALLENE OXIDE SYNTHASE 1* | Direct | GACGCATCATTCGAAATCAA |
|  |  |  | Reverse | GCGTTTTCAGTTTCCGACCC |
| *Solyc03g122190.2* | *JAZ 2* | *JASMONATE ZIM DOMAIN 2* | Direct | TAGCTCATGCAGTGGTGGT |
|  |  |  | Reverse | ATCCGTTGTTACCATCTTCTTC |
| *Solyc03g044820.2* | *JMT* | *JASMONIC ACID CARBOXYLMETHYL TRANSFERASE* | Direct | TTTACAACAACGGACAGGATA |
|  |  |  | Reverse | ACTCATCGAACAATGGTACG |
| *Solyc01g087260.2* | *CCD1b* | *CAROTENOID CLEAVAGE DIOXYGENASE 1b* | Direct | GATGCGAAAACAATGTCAGC |
|  |  |  | Reverse | ATGGTGCCAATTGAAAACAGA |
| *Solyc04g070980.2* | *CAS 1* | *CYCLOARTENOL SYNTHASE 1* | Direct | CGGTCCCGTATCATGAAGT |
|  |  |  | Reverse | GGTGTGGGTAGTAAAGGTCTTC |
| *Solyc05g051750.2* | *PS* | *PROSYSTEMIN* | Direct | CACCATGAGAAGGGAGGAGA |
|  |  |  | Reverse | CCTCCTCATGTTCCATCTTTG |
| *Solyc03g020060.2* | *PIN II* | *PROTEINASE INHIBITOR II* | Direct | GAAAATCGTTAATTTATCCCAC |
|  |  |  | Reverse | ACATACAAACTTTCCATCTTTA |
| *Solyc08g074680.2* | *PPO* | *POLYPHENOL OXIDASE* | Direct | TTGGCGGACCTTATGATCTC |
|  |  |  | Reverse | CATTGTTGTCCACGTTCAGG |
| *Solyc09g007910.2* | *PAL3* | *PHENYLALANINE AMMONIA LYASE 3* | Direct | GTTAGAATGCCTTAAGGAATGG |
|  |  |  | Reverse | GCTACAAACCAATATATTCAAGAG |
| *Solyc10g086180.1* | *PAL 4* | *PHENYLALANINE AMMONIA LYASE 4* | Direct | GTTGTGAATGCTTGTCTAGTGC |
|  |  |  | Reverse | ATAGACATAAGCACACTGTCAC |
| *Solyc03g042560.1* | *PAL 5* | *PHENYLALANINE AMMONIA LYASE 5* | Direct | GCTGAGCAACACAACCAAGA |
|  |  |  | Reverse | AGCAGATTGGAAGAGGAGCA |
| *Solyc05g005560.2* | *AROGP3* | *POLYGALACTURONASE NON-CATALYTIC SUBUNIT* | Direct | GACAACTATGGTGCCCCTG |
|  |  |  | Reverse | CTTCACCGCCATTTGCATC |
| *Solyc10g005840.2* | *FPS1* | *FARNESYL PYROPHOSPHATE SYNTHASE 1* | Direct | CATTCTTCTTCGCAACCACA |
|  |  |  | Reverse | CGGCGATGAATAGACAATGA |
| *Solyc06g084240.1* | *CPS* | *ENT-COPALYL DIPHOSPHATE SYNTHASE* | Direct | GAATTTGAGCAGGATTGGTGA |
|  |  |  | Reverse | TGTGCATGATGTCCTTTGGTA |
| *Solyc07g066670.2* | *KS* | *ENT-KAURENE SYNTHASE* | Direct | ACTGATTGCTGTTCGGAGAGA |
|  |  |  | Reverse | TGGCACCGACATATCTTTAGC |
| *Solyc10g046820.1* | *GA3ox* | *GA 3-OXIDASE* | Direct | AATATGAAAAGGAAATGGAAAAGC |
|  |  |  | Reverse | GGTGAAGGATTGTTAATATGGTAG |
| *Solyc01g090660.2* | *CCD7* | *CAROTENOID CLEAVAGE DIOXYGENASE 7* | Direct | AGCCAAGAATTCGAGATCCC |
|  |  |  | Reverse | GGAGAAAGCCCACATACTGC |
| *Solyc01g087250.2* | *CCD1a* | *CAROTENOID CLEAVAGE DIOXYGENASE 1a* | Direct | AAGCTTGAGAATTTCTGCA |
|  |  |  | Reverse | GCCTGTGTAGTTCTCGTTGAT |
| *Solyc01g087260.2* | *CCD1b* | *CAROTENOID CLEAVAGE DIOXYGENASE 1b* | Direct | GATGCGAAAACAATGTCAGC |
|  |  |  | Reverse | ATGGTGCCAATTGAAAACAGA |
| *Solyc07g043490.1* | *GAME 1* | *GLYCOALKALOID METABOLISM 1* | Direct | GCATTTTGGTCCGCTCTCTC |
|  |  |  | Reverse | GCGCATTCAACCAATCTACAAC |
| *Solyc03g113400.2* | *LHA* | *H^+^-ATPASE* | Direct | GCAAATACCGTCCTGGCATA, |
|  |  |  | Reverse | CCTGTTGAGCAAGACGATGA |
| *Solyc10g085570.1* | *CTR4* | *CONSTITUTIVE TRIPLE RESPONSE 4* | Direct | AGTGCTTTTCATGGGTGCTG |
|  |  |  | Reverse | ATCCCCTTTGCCACATCGTA |
| *Solyc01g095080.2* | *ACS2* | *1-AMINOCYCLOPROPANE-1-CARBOXYLIC ACID SYNTHASE 2* | Direct | TGATGGAACGGTTGATATTG |
|  |  |  | Reverse | TTAACGAACTAATGGTGAGGG |
| *Solyc08g008100.2* | *ACS6* | *1-AMINOCYCLOPROPANE-1-CARBOXYLIC ACID SYNTHASE 6* | Direct | GGATGATGCTACAATGAAGATT |
|  |  |  | Reverse | CGTACGATTAATTCCTTGCTTG |
| *Solyc02g081190.2* | *ACO4* | *1-AMINOCYCLOPROPANE-1-CARBOXYLIC ACID OXIDASE 4* | Direct | GCTCAACAAGATGGCACTAG |
|  |  |  | Reverse | TCTCCACAGCCTTCATTG |
| Solyc09g007870.2 | EIN 2 | *ETHYLENE INSENSITIVE 2* | Direct | TCTCTGGGCTTGCTAACCAT |
|  |  |  | Reverse | AGCCCATTTCCCAGGATC |
| *Solyc06g073730.1* | *EIN 3* | *ETHYLENE INSENSITIVE 3* | Direct | AGATTTCTTGACGTTTGGTG |
|  |  |  | Reverse | AGCCACCTCCACTTCCTT |
| *Solyc10g085230.1* | *ERF1b* | *ETHYLENE RESPONSE FACTOR 1b* | Forward | CATTGTGGTTGGAATTCTACG |
|  |  |  | Reverse | TTTCTCTATGACCGTTCTCTCG |
| *Solyc04g080960.2* | *CYP 2* | *CYSTEINE PROTEINASE 2* | Direct | CATCTGATTTTGACTGGCG |
|  |  |  | Reverse | GGTCACACTCATGGTCACAA |
| *Solyc06g083040.2* | *SCP* | *WOUND-INDUCIBLE SERINE CARBOXYPEPTIDASE* | Direct | GTGGACCTGGTTGTTCAAG |
|  |  |  | Reverse | ATGGATTGTTGTGCAGAGAA |
| *Solyc08g081690.2* | *RBOH1* | *RESPIRATORY BURST OXIDASE HOMOLOG 1 (NADPH OXIDASE)* | Direct | TTTTCCAAGACCTTAACCAAGC |
|  |  |  | Reverse | ACTTTGTTGTGCCCTTTTGGT |
| *Solyc01g106620.2* | *PR1* | *PATHOGENESIS-RELATED PROTEIN 1* | Direct | CAACCTATCTCTCCATAACCAAA |
|  |  |  | Reverse | TCTCCTAAGCAATTCCAACATAAT |
| *Solyc06g059710.2* | *SSI2* | *SA INSENSITIVITY OF npr1-5* | Direct | GGCCACTCATCTAATAAACTTT |
|  |  |  | Reverse | TTTTCTGGTGGCATTGAG |
| *Solyc09g091550.2* | *SAMT* | *SALICYLIC ACID METHYLTRANSFERASE* | Direct | CCAATAAGAGATCAAGCCATAAG |
|  |  |  | Reverse | TCATTTCCAGGGAGATCATT |
| *Solyc07g055280.2* | *WRKY65* | *WRKY TRANSCRIPTION FACTOR 65* | Direct | CAACTCTTTCTTAGCAAACTTTTTAT |
|  |  |  | Reverse | CGGTGGAATCTTCTTGTTTAT |
| *Solyc11g010850.1* | *DXS2* | *1-DEOXY-D-XYLULOSE-5-PHOSPHATE SYNTHASE 2* | Direct | AGACGGTCCAACGCATTGT |
|  |  |  | Reverse | CCTCTAGGAAATCGGAAACA |
| *Solyc02g082260.2* | *HMGR1* | *3-HYDROXY-3-METHYLGLUTARYL COENZYME A REDUCTASE 1* | Direct | GGATAAGAAGCCAGCAGCAG |
|  |  |  | Reverse | CCACCACCTACGGTACCAAC |
| *Solyc12g010980.1* | *BEAT* | *BENZYL ALCOHOL ACETYLTRANSFERASE* | Forward | CGCGGTAAGGTAGCAGATTT |
|  |  |  | Reverse | GAGTAGCTTTGACGCATTTATTG |
| *Solyc03g115810.2* | *SAND* | *SAND FAMILY PROTEIN* | Direct | TTTGCTTGGAGGAACAGAC |
|  |  |  | Reverse | GAATCAGCTACATCATGCAAGA |
| *Solyc10g049850.1* | *TIP4I* | *TIP 41-LIKE FAMILY PROTEIN* | Direct | GCTGCGTTTCTGGCTTAGG |
|  |  |  | Reverse | GACAAGGCCTGAAATGTGGT |
