# Supplementary material for: Distinct gene expression and secondary metabolite profiles in suppressor of prosystemin-mediated responses2 (spr2) tomato mutants having impaired mycorrhizal colonization
Source: PeerJ. 2020 Apr 16;8:e8888. doi: 10.7717/peerj.8888 (PMC7167247; doi:10.7717/peerj.8888)
Supplement: Supplemental Information 3 — The effect on growth, measured as differences in plant height, and in photosynthesis-related parameters, defined in terms of chlorophyll content and chlorophyll fluorescence emission, were determined at four different days post inoculation (dpi) in non-colonized (WT and spr2) and in mycorrhizal (WT-M and spr2-M) plants1. [file peerj-08-8888-s003.docx]

|  | **15 dpi** | **23 dpi** | **30 dpi** | **37 dpi** |
| --- | --- | --- | --- | --- |
|  | **Plant height (cm)** | | | |
| **WT** | 8.03 ± 0.79^a^ | 11.14 ± 0.89^b^ | 12.77 ± 0.83^b^ | 15.20 ± 1.62^ab^ |
| **WT-M** | 8.18 ± 0.82^a^ | 13.09 ± 1.25^a^ | 14.85 ± 1.41^a^ | 17.70 ± 2.31^ab^ |
| ***spr2*** | 4.69 ± 0.77^b^ | 8.36 ± 0.75^c^ | 9.56 ± 1.45^c^ | 13.61 ± 3.12^b^ |
| ***spr2*-M** | 5.11 ± 0.53^b^ | 7.54 ± 0.98^c^ | 9.13 ± 1.12^c^ | 12.65 ± 1.37^b^ |
|  | **Chlorophyll content** | | | |
| **WT** | ND | 34.20 ± 3.13^a^ | 43.82 ± 5.28^b^ | 59.57 ± 4.91^a^ |
| **WT-M** | ND | 25.88 ± 2.09^b^ | 47.00 ± 6.17^a^ | 49.45 ± 4.53^bc^ |
| ***spr2*** | ND | 20.70 ± 4.32^c^ | 25.37 ± 8.66^c^ | 40.31 ± 11.76^c^ |
| ***spr2*-M** | ND | 25.38 ± 3.69^b^ | 38.74 ± 7.76^b^ | 50.07 ± 9.92^b^ |
|  | **Chlorophyll fluorescence** | | | |
|  | **Fv/ Fw** | | | |
| **WT** | ND | 0.82 ± 0.005^b^ | ND | 0.80 ± 0.023^b^ |
| **WT-M** | ND | 0.82 ± 0.000^b^ | ND | 0.81 ± 0.008^ab^ |
| ***spr2*** | ND | 0.84 ± 0.005^a^ | ND | 0.83 ± 0.024^ab^ |
| ***spr2*-M** | ND | 0.83 ± 0.012^a^ | ND | 0.84 ± 0.005^b^ |
|  | **PI_abs_** | | | |
| **WT** | ND | 4.28 ± 0.43^a^ | 4.18 ± 0.48^b^ | 3.87 ± 0.51^b^ |
| **WT-M** | ND | 3.78 ± 0.21^a^ | 4.89 ± 0.56^ab^ | 4.04 ± 0.60^b^ |
| ***spr2*** | ND | 4.03 ± 0.96^a^ | 5.27 ± 0.88^ab^ | 5.86 ± 0.65^a^ |
| ***spr2*-M** | ND | 4.67 ± 0.61^a^ | 5.89 ± 1.39^a^ | 6.35 ± 0.31^a^ |

^1^Data were compiled from plants corresponding to the E2 experiment in which colonization efficiency in roots inoculated with a six AMF species consortium, was determined 45 dpi.
