# Supplementary material for: Distinct gene expression and secondary metabolite profiles in suppressor of prosystemin-mediated responses2 (spr2) tomato mutants having impaired mycorrhizal colonization
Source: PeerJ. 2020 Apr 16;8:e8888. doi: 10.7717/peerj.8888 (PMC7167247; doi:10.7717/peerj.8888)
Supplement: Supplemental Information 5 — The ions listed were generated as part of an untargeted DLI-ESI-MS analysis. They underwent significant differences in abundance in mycorrhizal roots of wild-type (WT) and mutant spr2 tomato plants. [file peerj-08-8888-s005.docx]

|  | **Increased by AMF^1^** | | | | **Decreased by AMF^2^** | | | |  |
| --- | --- | --- | --- | --- | --- | --- | --- | --- | --- |
|  | **BOTH** | |  |  | **BOTH** | |  |  |  |
|  | **WT** | ***spr2*** | **WT only** | ***spr2* only** | **WT** | ***spr2*** | **WT only** | ***spr2* only** |  |
| ***m*/*z*^3^** |  |  |  |  |  |  |  |  | **Putative metabolite** |
| 84.15 |  |  |  |  |  |  |  |  | 3-methyl-2-butenal; 1-penten-3-one,trans-2-pentenal |
| 100.07 |  |  |  |  |  |  |  |  | Hydantoin, 2-aminobut-2-enoate |
| 104.21 |  |  |  |  |  |  |  |  | Hydroxypyruvate; 3-methylthiopropanal, choline, cadaverine |
| 140.05 |  |  |  |  |  |  |  |  | ethyleneglycol-P, phosphoryl-ethanolamine |
| 177.32 |  |  |  |  |  |  |  |  | Indole-3-glycol, 4-methoxy-3-indolylmethylamine, L-dihomomethionine; N-carbamoyl-L-aspartate, (2R (2S)-2-isopropylmalate, L-ascorbate, allantoate, herniarin, N-hydroxyl-tryptamine, |
| 203.36 |  |  |  |  |  |  |  |  | 1,4-dihydroxy-2-naphthoate, N6-dimethylallyladenine, 2-oxo-8-methylthiooctanoate, 9-methylthiononanaldoxime, 4-amino-2-methyl-5, phosphomethylpyrimidine, indole-3-pyruvate, 8-(methylsulfanyl)-2-oxooctanoate, 2IP, (1E)-N-hydroxy-9-(methylsulfanyl)-1-nonanimine, vernolate, methyl N-acetyl-6-ammonia-L-norleucinate, bergaptol, indole-3-butyrate, (-)-cuparene, 1-chloro-2-thermospermine, spermine |
| 307.69 |  |  |  |  |  |  |  |  | 2'-Deoxy-5'-cytidylic acid, glutathione, gallocatechin, leucocyanidin |
| 317.19 |  |  |  |  |  |  |  |  | Myricetin; GA9 (GA4 precursor)-GA12 aldehyde |
| 330.24 |  |  |  |  |  |  |  |  | 3',5'-cyclic IMP; GA9 (GA4 precursor )-GA12 aldehyde |
| 335.75 |  |  |  |  |  |  |  |  | Caffeoylshikimate, N4-(β-N-acetyl-D-glucosaminyl)-L-asparagine, isopentenyl adenosine |
| 365.6 |  |  |  |  |  |  |  |  | indole-3-butyryl-glucose, isopentenyladenine-7-N-glucoside, isopentenyladenine-9-N-glucoside, 8-methylthiooctylhydroximoyl-cysteinylglycine, (15Z)-tetracosenoate |
| 366.86 |  |  |  |  |  |  |  |  | D-sedoheptulose-1,7-bisphosphate, N-acetyl-farnesylcysteine |
| 368.88 |  |  |  |  |  |  |  |  | O-feruloylquinate |
| 369.62 |  |  |  |  |  |  |  |  | acetylajmaline |
| 370.72 |  |  |  |  |  |  |  |  | sinapaldehyde glucoside, (+)-sesamolin |
| 372.86 |  |  |  |  |  |  |  |  | syringin, (+)-sesamolinol, 7-O-acetylsalutaridinol, 1,2-dioctanoyl-3-methyl-1,2,3-butanetriol |
| 377.75 |  |  |  |  |  |  |  |  | 8-oxo-GMP, Kinetin-9-N-glucoside, kinetin-7-N-glucoside |
| 381.12 |  |  |  |  |  |  |  |  | Trans, trans-farnesyl diphosphate |
| 383.85 |  |  |  |  |  |  |  |  | dihydrozeatin-9-N-glucoside, dihydrozeatin-O-glucoside, dihydrozeatin-7-N-glucose |
| 384.87 |  |  |  |  |  |  |  |  | dCDP, S-adenosyl-L-homocysteine, 7-dehydrocholesterol, desmosterol, 5α-cholesta-7,24-dien-3β-ol, zymosterol, cholest-5-en-3-one, 5α-cholesta-8-en-3-one, 7-dehydrocholesterol |
| 398.52 |  |  |  |  |  |  |  |  | isorhamnetin 3-sulfate, 5,5'-dithio-bis-2-nitrobenzoate, 4α-methyl-5α-cholesta-8-en-3-one, 24-methylenecholesterol, 24-methyldesmosterol, campest-4-en-3-one, 4α-methyl-5α-cholest-7-en-3-one, 4α-methyl-5α-cholesta-7,24-dien-3β-ol, brassicasterol, campest-5-en-3-one, crinosterol, episterol, 4α-methyl-zymosterol, fecosterol |
| 415.71 |  |  |  |  |  |  |  |  | 17-O-deacetylvindoline |
| 417.32 |  |  |  |  |  |  |  |  | NI^4^ |
| 429.18 |  |  |  |  |  |  |  |  | trans-zeatin riboside monophosphate, cis-zeatin riboside monophosphate, 4α-carboxy-5α-cholesta-8-en-3β-ol, cis-zeatin riboside monophosphate |
| 439.66 |  |  |  |  |  |  |  |  | phenylacetohydroximoyl-glutathione |
| 440.47 |  |  |  |  |  |  |  |  | GDP, 4α-formyl-stigmasta-7,24(241)-dien-3β-ol, 14-oxolanosterol, 4α-formyl,4β,14α-dimethyl-9β,19-cyclo-5α-cholest-24-en-3β-ol, demethylphylloquinol, 24-methylenecycloartanol |
| 441.61 |  |  |  |  |  |  |  |  | GDP group, 7,8-dihydrofolate, 4α-carboxy-4β-methyl-5α-cholesta-8,24-dien-3β-ol, 4α-carboxy-ergosta-7,24(241)-dien-3β-ol |
| 443.79 |  |  |  |  |  |  |  |  | Tetrahydrofolate, 4α-carboxy-4β-methyl-5α-cholesta-8-en-3β-ol, 3β-hydroxy-4β-methyl-5α-cholest-7-ene-4α-carboxylate |
| 444.87 |  |  |  |  |  |  |  |  | Arabidiol, lupan-3β,20-diol, 4,4-dimethyl-14α-hydroxymethyl-5α-cholesta-8-en-3β-ol |
| 457.17 |  |  |  |  |  |  |  |  | 5-methyl-tetrahydrofolate |
| 463.69 |  |  |  |  |  |  |  |  | dCTP, quercetin-3-glucoside, quercetin 4'-O-glucoside, quercetin 7-O-glucoside, quercetin 3'-O-glucoside |
| 467.77 |  |  |  |  |  |  |  |  | NI |
| 469.75 |  |  |  |  |  |  |  |  | 4α-carboxy-4β,14α-dimethyl-9β,19-cyclo-5α-ergost-24(241)-en-3β-ol |
| 478.92 |  |  |  |  |  |  |  |  | dTTP, indole-3-acetohydroximoyl-glutathione, (22R,23R)-28-homocastasterone |
| 485.73 |  |  |  |  |  |  |  |  | NI |
| 488.03 |  |  |  |  |  |  |  |  | Ditp |
| 489.77 |  |  |  |  |  |  |  |  | β-D-glucosyl crocetin |
| 505.79 |  |  |  |  |  |  |  |  | NI |
| 513.06 |  |  |  |  |  |  |  |  | NI |
| 521.26 |  |  |  |  |  |  |  |  | NI |
| 529.46 |  |  |  |  |  |  |  |  | NI |
| 537.39 |  |  |  |  |  |  |  |  | NI |
| 542.8 |  |  |  |  |  |  |  |  | 15,9'-di-cis-phytofluene, all-trans phytofluene, (9cis,15cis)-7,7',8,8',11',12'-Hexahydro-psi,psi-carotene |
| 545.42 |  |  |  |  |  |  |  |  | dihydrozeatin-9-N-glucoside-O-glucoside, 3-hexaprenyl-4-hydroxybenzoate, prephytoene diphosphate, (all-E)-Phytoene |
| 549.86 |  |  |  |  |  |  |  |  | delphinidin 3-O-(6''-O-malonyl)-β-glucoside |
| 551.12 |  |  |  |  |  |  |  |  | NI |
| 552.39 |  |  |  |  |  |  |  |  | Zeinoxanthin, β-carotene 15,15' epoxide, ε,ε-carotene-3-diol, 1'-hydroxy-γ-carotene, β-cryptoxanthin, 1'-hydroxy-γ-carotene, zeinoxanthin, β-carotene 15,15' epoxide, β-cryptoxanthin, ε,ε-carotene-3-diol, beta-cryptoxanthin, (3S)-beta,beta-Caroten-3-ol |
| 553.63 |  |  |  |  |  |  |  |  | NI |
| 555.8 |  |  |  |  |  |  |  |  | 1-(5-phospho-β-D-ribosyl)-AMP |
| 558.68 |  |  |  |  |  |  |  |  | NI |
| 561.85 |  |  |  |  |  |  |  |  | 3,4-dihydroxy-5-all-trans-hexaprenylbenzoate |
| 565.91 |  |  |  |  |  |  |  |  | Canthaxanthin |
| 566.39 |  |  |  |  |  |  |  |  | NI |
| 569.82 |  |  |  |  |  |  |  |  | D-myo-inositol (1,2,3,5,6) pentakisphosphate, D-myo-inositol (1,2,3,4,5)-pentakisphosphate, 1D-myo-inositol (1,2,3,4,6)-pentakisphosphate, D-myo-inositol (1,2,4,5,6)-pentakisphosphate, D-myo-inositol 1,3,4,5,6-pentakisphosphate, 2-dipalmitoylglycerol |
| 571.85 |  |  |  |  |  |  |  |  | D-myo-inositol pentakisphosphate ISOMERS |
| 573.8 |  |  |  |  |  |  |  |  | phosphoribulosylformimino-AICAR-P, 1-(5-phospho-β-D-ribosyl)-5-[(5-phosphoribosylamino)methylideneamino]imidazole-4-carboxamide, THF-L-glutamate, (22R,23R)-28-homobrassinolide-22-sulfate, 3,3'-[(Ethylimino)bis(methylene)]bis(5,5-diphenyl-2,4-imidazolidinedione), 3,3'-[(Ethylimino)bis(methylene)]bis(5,5-diphenyl-2,4-imidazolidinedione) |
| 575.51 |  |  |  |  |  |  |  |  | 3-methoxy-4-hydroxy-5-all-trans-hexaprenylbenzoate, THF-L-glutamate |
| 576.89 |  |  |  |  |  |  |  |  | solasodine 3-O-β-D-glucopyranoside, 3-O-β-D-glucosyl-β-sitosterol |
| 578.93 |  |  |  |  |  |  |  |  | pelargonidin-3-O-rutinoside, kaempferol-3-rhamnoside-7-rhamnoside, apigenin 7-O-neohesperidoside, pelargonidin 3-O-β-D-p, coumaroylglucoside, kaempferitrin, rhoifolin |
| 581.85 |  |  |  |  |  |  |  |  | NI |
| 585.81 |  |  |  |  |  |  |  |  | GDP-4-dehydro-6-deoxy-α-D-mannose, bilirubin, (3Z)-phytochromobilin, antheraxanthin |
| 587.81 |  |  |  |  |  |  |  |  | GDP-β-L-fucose, ADP-α-D-glucose, dTDP-N-acetylthomosamine, [(2R,3S,4R,5R)-5-(2-Amino-6-oxo-1,6-dihydro-9H-purin-9-yl)-3,4 dihydroxytetrahydro-2-furanyl]methyl (2R,3S,4R,6R)-3,4-dihydroxy-6-methyl-5-oxotetrahydro-2H-pyran-2-yl dihydrogen diphosphate  [[(2R,3S,4R,5R)-5-(6-aminopurin-9-yl)-3,4-dihydroxy-tetrahydrofuran-2-yl]methoxy-oxido-phosphoryl] [(2R,3R,4S,5S,6R)-3,4,5-trihydroxy-6-(hydroxymethyl)tetrahydropyran-2-yl] phosphate, all-trans-hexaprenyl diphosphate, presqualene diphosphate |
| 589.74 |  |  |  |  |  |  |  |  | trans-zeatin riboside triphosphate |
| 591.85 |  |  |  |  |  |  |  |  | pheophorbide a |
| 593.76 |  |  |  |  |  |  |  |  | 2-carboxylate-4-methyl-5-beta-(ethyl adenosine 5-diphosphate) thiazole, pheophorbide A |
| 597.84 |  |  |  |  |  |  |  |  | 2-phospho-4-(cytidine 5'-diphospho)-2-C-methyl-D-erythritol |
| 599.86 |  |  |  |  |  |  |  |  | magnesium-protoporphyrin IX 13-monomethyl ester |
| 601.8 |  |  |  |  |  |  |  |  | 5'-O-[({[(2R,3S)-2,4-Dihydroxy-3-methyl-3-(phosphonooxy)butoxy](hydroxy)phosphoryl}oxy)(hydroxy) phosphoryl] cytidine, trans-neoxanthin, violaxanthin, 9'-cis-neoxanthin, 9-cis-violaxanthin |
| 603.75 |  |  |  |  |  |  |  |  | GDP-α-D-glucose, GDP-β-L-gulose, GDP-β-L-galactose, GDP-α-D-mannose |
| 605.8 |  |  |  |  |  |  |  |  | UDP-N-acetyl-α-D-glucosamine, UDP-N-acetyl-α-D-galactosamine, pheophorbide b, GDP-α-D-glucose, GDP-α-D-mannose |
| 607.8 |  |  |  |  |  |  |  |  | UDP-N-acetyl-α-D-glucosamine, UDP-N-acetyl-β-D-galactosamine |
| 609.87 |  |  |  |  |  |  |  |  | kaempferol 3-O-β-D-glucosyl-(1->2)-glucoside |
| 613.82 |  |  |  |  |  |  |  |  | 3-heptaprenyl-4-hydroxybenzoate, 13-hydroxy-magnesium-protoporphyrin IX 13-monomethyl ester, glutathione disulfide |
| 616.49 |  |  |  |  |  |  |  |  | NI |
| 617.77 |  |  |  |  |  |  |  |  | protoheme IX |
| 621.81 |  |  |  |  |  |  |  |  | ADP ribose 1'',2''-cyclic phosphate |
| 623.7 |  |  |  |  |  |  |  |  | leukotriene-C4 |
| 625.62 |  |  |  |  |  |  |  |  | NI |
| 627.71 |  |  |  |  |  |  |  |  | UDP-α-D-sulfoquinovopyranose, chitotriose, chlorophyllide b |
| 631.8 |  |  |  |  |  |  |  |  | tricaffeoyl spermidine |
| 637.8 |  |  |  |  |  |  |  |  | NI |
| 641.78 |  |  |  |  |  |  |  |  | NI |
| 643.51 |  |  |  |  |  |  |  |  | 3-methoxy-4-hydroxy-5-all-trans-heptaprenylbenzoate |
| 649.79 |  |  |  |  |  |  |  |  | NI |
| 651.82 |  |  |  |  |  |  |  |  | β-D-gentiobiosyl crocetin, all-trans-heptaprenyl diphosphate |
| 653.78 |  |  |  |  |  |  |  |  | NI |
| 657.19 |  |  |  |  |  |  |  |  | Myricetin 3-O-(4''-O-acetyl-2''-O-galloyl)-alpha-L-rhamnopyranoside |
| 659.2 |  |  |  |  |  |  |  |  | 1D-myo-inositol 1,2,3,4,5,6-hexakisphosphate |
| 661.71 |  |  |  |  |  |  |  |  | NI |
| 667.81 |  |  |  |  |  |  |  |  | Stachyose, maltotetraose |
| 669.19 |  |  |  |  |  |  |  |  | NI |
| 675.8 |  |  |  |  |  |  |  |  | NI |
| 677.76 |  |  |  |  |  |  |  |  | PppGpp |
| 679.79 |  |  |  |  |  |  |  |  | NI |
| 681.81 |  |  |  |  |  |  |  |  | (S)-NADH+, (R)-NADH+ |
| 683.74 |  |  |  |  |  |  |  |  | mono-trans, poly-cis-decaprenyl group, 3-octaprenyl-4-hydroxybenzoate |
| 685.9 |  |  |  |  |  |  |  |  | 3'-dephospho-CoA |
| 691.18 |  |  |  |  |  |  |  |  | NI |
| 693.71 |  |  |  |  |  |  |  |  | curcumin 4'-O-β-D-gentiobioside, curcumin diglucoside |
| 695.81 |  |  |  |  |  |  |  |  | curcumin diglucoside |
| 697.36 |  |  |  |  |  |  |  |  | NI |
| 699.74 |  |  |  |  |  |  |  |  | 2-octaprenyl-3-methyl-6-methoxyquinol, tetrahydropteroyl tri-L-glutamate |
| 703.82 |  |  |  |  |  |  |  |  | NI |
| 707.16 |  |  |  |  |  |  |  |  | NI |
| 710.02 |  |  |  |  |  |  |  |  | NI |
| 711.26 |  |  |  |  |  |  |  |  | NI |
| 713.23 |  |  |  |  |  |  |  |  | 5-methyltetrahydropteroyl tri-L-glutamate |
| 714.95 |  |  |  |  |  |  |  |  | NI |
| 715.74 |  |  |  |  |  |  |  |  | 3-demethylubiquinol-8 |
| 719.8 |  |  |  |  |  |  |  |  | prephytoene diphosphate, all-trans-octaprenyl diphosphate |
| 723.85 |  |  |  |  |  |  |  |  | 2-nonaprenyl-6-hydroxyphenol, all-trans-octaprenyl diphosphate |
| 725.61 |  |  |  |  |  |  |  |  | NI |
| 729.55 |  |  |  |  |  |  |  |  | ubiquinol-8 |
| 731.8 |  |  |  |  |  |  |  |  | NI |
| 733.78 |  |  |  |  |  |  |  |  | L-1-glycero-3-phosphocholine |
| 736.98 |  |  |  |  |  |  |  |  | 2-methyl-6-solanesyl-1,4-benzoquinol, dihydroxyferuloyl-sinapoyl spermidine |
| 741.85 |  |  |  |  |  |  |  |  | NADPH, 1-18:3-2-trans-16:1-phosphatidylglycerol |
| 744.06 |  |  |  |  |  |  |  |  | 1-18:2-2-trans-16:1-phosphatidylglycerol, 1-18:3-2-16:0-phosphatidylglycerol |
| 745.86 |  |  |  |  |  |  |  |  | 1-18:1-2-trans-16:1-phosphatidylglycerol, 1-18:2-2-16:0-phosphatidylglycerol |
| 747.83 |  |  |  |  |  |  |  |  | 1-18:3-2-16:3-monogalactosyldiacylglycerol |
| 752.91 |  |  |  |  |  |  |  |  | 1-18:3-2-16:1-monogalactosyldiacylglycerol, 1-18:2-2-16:2-monogalactosyldiacylglycerol, 1-18:1-2-16:3-monogalactosyldiacylglycerol, plastoquinol-9, all-trans-undecaprenyl group, di-trans,octa-cis-undecaprenyl group, zaragozic acid C, 1-18:1-2-16:0-phosphatidylglycerol, plastoquinol-9 |
| 756.51 |  |  |  |  |  |  |  |  | 1-16:0-2-18:3-phosphatidylcholine, kaempferol 3-O-rhamnosyl(1->2)glucoside-7-O-rhamnoside, kaempferol-3-O-gentiobioside-7-O-rhamnoside, shisonin, bisdemalonylsalvianin, pelargonidin-3,5-diglucoside-5-O-caffeoylglucoside, 1-18:2-2-16:1-monogalactosyldiacylglycerol, 1-18:3-2-16:0-monogalactosyldiacylglycerol, 1-18:1-2-16:2-monogalactosyldiacylglycerol, 1-18:2-2-16:0-monogalactosyldiacylglycerol, 1-18:1-2-16:1-monogalactosyldiacylglycerol, 1-18:1-2-16:0-monogalactosyldiacylglycerol |
| 758.85 |  |  |  |  |  |  |  |  | 1-palmitoyl-2-linoleoyl-phosphatidylcholine, 1-18:3-2-16:3-monogalactosyldiacylglycerol, 1-18:2-2-16:3-monogalactosyldiacylglycerol, 1-18:3-2-16:2-monogalactosyldiacylglycerol, 1-18:3-2-16:1-monogalactosyldiacylglycerol, 1-18:2-2-16:2-monogalactosyldiacylglycerol  1-18:1-2-16:3-monogalactosyldiacylglycerol, 1-18:2-2-16:1-monogalactosyldiacylglycerol, 1-18:3-2-16:0-monogalactosyldiacylglycerol  1-18:1-2-16:2-monogalactosyldiacylglycerol, 1-18:2-2-16:0-monogalactosyldiacylglycerol, 1-18:1-2-16:1-monogalactosyldiacylglycerol  1-18:1-2-16:0-monogalactosyldiacylglycerol |
| 769.81 |  |  |  |  |  |  |  |  | 4,8-sphingadienine |
| 773.72 |  |  |  |  |  |  |  |  | quercetin 3-O-rhamnosyl(1->2)glucoside-7-O-rhamnoside, quercetin 3-O-gentiobioside-7-O-rhamnoside |
| 775.83 |  |  |  |  |  |  |  |  | NI |
| 778.11 |  |  |  |  |  |  |  |  | 1-18:3-2-18:3-phosphatidylcholine |
| 780.83 |  |  |  |  |  |  |  |  | 1-18:2-2-18:3-phosphatidylcholine, 1-18:3-2-18:2-phosphatidylcholine, 3-methoxy-4-hydroxy-5-all-trans-nonaprenylbenzoate |
| 782.83 |  |  |  |  |  |  |  |  | 1-18:2-2-18:2-sn-glycerol-3-phosphocholine, 1-18:3-2-18:1-phosphatidylcholine, 1-18:1-2-18:3-phosphatidylcholine, FAD, |
| 785.85 |  |  |  |  |  |  |  |  | FADH2, FAD, 1-18:3-2-18:3-phosphatidylcholine, 1-18:2-2-18:3-phosphatidylcholine, 1-18:3-2-18:2-phosphatidylcholine, 1-18:2-2-18:2-sn-glycerol-3-phosphocholine, 1-18:3-2-18:1-phosphatidylcholine, 1-18:1-2-18:3-phosphatidylcholine, 1-18:2-2-18:1-phosphatidylcholine, 1-18:1-2-18:2-phosphatidylcholine, 1-18:1-2-18:1-sn-glycerol-3-phosphocholine |
| 787.8 |  |  |  |  |  |  |  |  | FADH2 |
| 789.85 |  |  |  |  |  |  |  |  | all-trans-nonaprenyl diphosphate, methionyl peptide |
| 791.8 |  |  |  |  |  |  |  |  | 1-18:3-2-18:3-monogalactosyldiacylglycerol, formyl-CoA, all-trans-nonaprenyl diphosphate |
| 796.5 |  |  |  |  |  |  |  |  | phosphatidylglycerophosphate (dioctadec-9-enoyl(Z)), zaragozic acid C, ubiquinol-9, 1-18:3-2-18:3-monogalactosyldiacylglycerol, 1-18:2-2-18:3-monogalactosyldiacylglycerol, 1-18:3-2-18:2-monogalactosyldiacylglycerol, 1-18:2-2-18:2-monogalactosyldiacylglycerol |
| 802.55 |  |  |  |  |  |  |  |  | NI |
| 804.57 |  |  |  |  |  |  |  |  | NI |
| 810.79 |  |  |  |  |  |  |  |  | luteolin 7-O-[β-D-glucuronosyl-(1,2)-β-D-glucuronide]-4'-O-β-D-glucuronide |
| 813.28 |  |  |  |  |  |  |  |  | NI |
| 815.44 |  |  |  |  |  |  |  |  | NI |
| 818.07 |  |  |  |  |  |  |  |  | 3-decaprenyl-4-hydroxybenzoate |
| 820.49 |  |  |  |  |  |  |  |  | carboxymethyl-CoA |
| 824.68 |  |  |  |  |  |  |  |  | phosphatidylglycerophosphate (1-octadecenoyl (9Z), 2-palmitoyl) |
| 826.75 |  |  |  |  |  |  |  |  | NI |
| 831.71 |  |  |  |  |  |  |  |  | methylacrylyl-CoA, crotonyl-CoA, UDP-3-O-[(3R)-3-hydroxymyristoyl]-N-acetyl-α-D-glucosamine |
| 832.76 |  |  |  |  |  |  |  |  | NI |
| 834.55 |  |  |  |  |  |  |  |  | oxalyl-CoA, 6-methoxy-3-methyl-2-all-trans-decaprenyl-1,4-benzoquinol |
| 836.73 |  |  |  |  |  |  |  |  | 3-hydroxypropanoyl-CoA, heme o, uroporphyrinogen-I, uroporphyrinogen-III |
| 841.43 |  |  |  |  |  |  |  |  | Malonylshisonin, monodemalonylsalvianin |
| 843.62 |  |  |  |  |  |  |  |  | Monodemalonylsalvianin |
| 850.44 |  |  |  |  |  |  |  |  | precorrin-1, 3-demethylubiquinol-10 |
| 851.68 |  |  |  |  |  |  |  |  | acetoacetyl-CoA, isovaleryl-CoA, 2-methylbutanoyl-CoA |
| 852.85 |  |  |  |  |  |  |  |  | malonyl-CoA, (R)-3-hydroxybutanoyl-CoA, (S)-3-hydroxybutanoyl-CoA |
| 854.63 |  |  |  |  |  |  |  |  | malonyl-CoA, (R)-3-hydroxybutanoyl-CoA, (S)-3-hydroxybutanoyl-CoA, hydroxymethylbilane |
| 855.71 |  |  |  |  |  |  |  |  | all-trans-decaprenyl diphosphate |
| 857.39 |  |  |  |  |  |  |  |  | precorrin-2, all-trans-decaprenyl diphosphate |
| 859.84 |  |  |  |  |  |  |  |  | all-trans-decaprenyl diphosphate |
| 861.66 |  |  |  |  |  |  |  |  | 2-methylacetoacetyl-CoA, β-ketovaleryl-CoA, hexanoyl-CoA |
| 862.61 |  |  |  |  |  |  |  |  | succinyl-CoA, sirohydrochlorin |
| 863.16 |  |  |  |  |  |  |  |  | 3-hydroxyisovaleryl-CoA, 2-methyl-3-hydroxybutyryl-CoA. Sirohydrochlorin |
| 866.61 |  |  |  |  |  |  |  |  | precorrin-2, benzoyl-CoA |
| 871.63 |  |  |  |  |  |  |  |  | pheophytin a, benzoyl-CoA |
| 875.68 |  |  |  |  |  |  |  |  | UDP-N-acetylmuramoyl-L-alanyl--D-glutamate, 3-oxohexanoyl-CoA |
| 877.72 |  |  |  |  |  |  |  |  | (S)-3-hydroxyhexanoyl-CoA |
| 879.58 |  |  |  |  |  |  |  |  | (E)-glutaconyl-CoA, itaconyl-CoA, 3-oxohexanoyl-CoA, UDP-N-acetylmuramoyl-L-alanyl--D-glutamate |
| 881.65 |  |  |  |  |  |  |  |  | glutaryl-CoA, (S)-3-hydroxyhexanoyl-CoA |
| 891.61 |  |  |  |  |  |  |  |  | tetrahydrogeranylgeranyl-chlorophyll a, trans-oct-2-enoyl-CoA, protochlorophyll a |
| 892.59 |  |  |  |  |  |  |  |  | chlorophyll a, 3-methylglutaconyl-CoA, octanoyl-CoA, trans-oct-2-enoyl-CoA |
| 897.44 |  |  |  |  |  |  |  |  | N-methylanthraniloyl-CoA, 6-O-mycolyl-trehalose 6-phosphate, (E)-cinnamoyl-CoA |
| 899.93 |  |  |  |  |  |  |  |  | N-methylanthraniloyl-CoA |
| 901.68 |  |  |  |  |  |  |  |  | geranylgeranyl-chlorophyll b, 2-trans-nonenoyl-CoA, N-methylanthraniloyl-CoA |
| 903.1 |  |  |  |  |  |  |  |  | 3-oxooctanoyl-CoA |
| 905.3 |  |  |  |  |  |  |  |  | (S)-3-hydroxyoctanoyl-CoA |
| 915.68 |  |  |  |  |  |  |  |  | 1-18:3-2-16:0-digalactosyldiacylglycerol, 1-16:0-2-18:3-digalactosyldiacylglycerol, 2-trans-decenoyl-CoA, trans-Δ2-decenoyl-CoA, 3-trans-decenoyl-CoA, 3-hydroxy-3-phenylpropionyl-CoA, P(1),P(5)-di(adenosine-5'-)pentaphosphate, 1-18:3-2-16:0-digalactosyldiacylglycerol, 1-16:0-2-18:3-digalactosyldiacylglycerol, 1-18:2-2-16:0-digalactosyldiacylglycerol, 1-16:0-2-18:2-digalactosyldiacylglycerol, 1-18:1-2-16:0-digalactosyldiacylglycerol |
| 917.6 |  |  |  |  |  |  |  |  | 1-18:2-2-16:0-digalactosyldiacylglycerol, 1-16:0-2-18:2-digalactosyldiacylglycerol, decanoyl-CoA, trans-2, cis-4-decadienoyl-CoA |
| 919.21 |  |  |  |  |  |  |  |  | 1-18:1-2-16:0-digalactosyldiacylglycerol, 3-hydroxy-nonanoyl-CoA, trans-2-decenoyl-CoA, 3-trans-decenoyl-CoA |
| 921.44 |  |  |  |  |  |  |  |  | Siroheme, decanoyl-CoA |
| 923.62 |  |  |  |  |  |  |  |  | NI |
| 925.76 |  |  |  |  |  |  |  |  | 3-(4-hydroxyphenyl)-3-oxo-propionyl-CoA, caffeoyl-CoA, 3-(4-hydroxyphenyl)-3-oxo-propionyl-CoA |
| 927.74 |  |  |  |  |  |  |  |  | 2-trans, 4-cis-undecadienoyl-CoA, 2-trans, 4-trans-undecadienoyl-CoA |
| 929.50 |  |  |  |  |  |  |  |  | 2-trans-undecenoyl-CoA, 4-cis-undecenoyl-CoA, 4-trans-undecenoyl-CoA, 3-trans-undecenoyl-CoA, 3-(4-hydroxyphenyl)-3-hydroxy-propionyl-CoA  caffeoyl-CoA, salvianin, 2-trans-undecenoyl-CoA 4-trans-undecenoyl-CoA, 3-trans-undecenoyl-CoA |
| 934.1 |  |  |  |  |  |  |  |  | (S)-3-hydroxydecanoyl-CoA, 3-hydroxy-decanoyl-CoA, (R)-3-hydroxydecanoyl-CoA, 2-trans-decenoyl-CoA, 3-oxodecanoyl-CoA |
| 937.76 |  |  |  |  |  |  |  |  | 1-18:3-2-18:3-digalactosyldiacylglycerol, (S)-3-hydroxydecanoyl-CoA, (R)-3-hydroxydecanoyl-CoA, 1-18:3-2-18:3-digalactosyldiacylglycerol, 1-18:2-2-18:3-digalactosyldiacylglycerol, 1-18:3-2-18:2-digalactosyldiacylglycerol, 1-18:2-2-18:2-digalactosyldiacylglycerol |
| 939.84 |  |  |  |  |  |  |  |  | 1-18:2-2-18:3-digalactosyldiacylglycerol, 1-18:3-2-18:2-digalactosyldiacylglycerol, feruloyl-CoA |
| 947.74 |  |  |  |  |  |  |  |  | 3-hydroxy-undecanoyl-CoA, 3-cis-dodecenoyl-CoA, 2-trans-dodecenoyl-CoA |
| 951.64 |  |  |  |  |  |  |  |  | delphinidin 3-O-glucosyl-5-O-(caffeoylglucoside-3'-O-glucoside), lauroyl-CoA |
| 953.43 |  |  |  |  |  |  |  |  | Indole-3-butyryl-CoA, 1,4-dihydroxy-2-naphthoyl-CoA, CDP-1,2-dipalmitoylglycerol |
| 955.51 |  |  |  |  |  |  |  |  | 5-hydroxy-feruloyl-CoA, 6'-hydroxyferuloyl-CoA, jasmonoyl-CoA, 2-trans-6-trans-tridecadienoyl-CoA, 6-cis, 2-trans-tridecadienoyl-CoA |
| 959.77 |  |  |  |  |  |  |  |  | 6'-hydroxyferuloyl-CoA, 3-hydroxy-5-trans-dodecenoyl-CoA, 3-oxododecanoyl-CoA, 6'-hydroxyferuloyl-CoA |
| 961.74 |  |  |  |  |  |  |  |  | NI |
| 963.81 |  |  |  |  |  |  |  |  | 3-oxododecanoyl-CoA |
| 965.82 |  |  |  |  |  |  |  |  | 3-keto-indole-3-butyryl-CoA |
| 967.79 |  |  |  |  |  |  |  |  | 2-trans, 5-cis, 7-trans-tetradecatrienoyl-CoA, 3-hydroxy-indole-3-butyryl-CoA |
| 973.72 |  |  |  |  |  |  |  |  | 3-hydroxy, 6-cis-tridecenoyl-CoA, 3-hydroxy, 6-trans-tridecenoyl-CoA, myristoyl-CoA, sinapoyl-CoA |
| 975.65 |  |  |  |  |  |  |  |  | NI |
| 977.69 |  |  |  |  |  |  |  |  | myristoyl-CoA |
| 979.75 |  |  |  |  |  |  |  |  | CDP-1-18:1(9Z)-2-16:0-glycerol |
| 981.74 |  |  |  |  |  |  |  |  | OPC4-trans-2-enoyl-CoA |
| 987.69 |  |  |  |  |  |  |  |  | 3-oxo-myristoyl-CoA |
| 989.87 |  |  |  |  |  |  |  |  | Maltohexaose, ajugose |
| 999.59 |  |  |  |  |  |  |  |  | 3-[(3aS,4S,5R,7aS)-5-hydroxy-7a-methyl-1-oxo-octahydro-1H-inden-4-yl]-3-oxopropanoyl-CoA, OPC4-3-hydroxyacyl-CoA, palmitoleyl-CoA |
| 1016.76 |  |  |  |  |  |  |  |  | curcumin 4'-O-β-D-gentiotetraside, curcumin 4',4''-O-β-D-digentiobioside |
| 1018.01 |  |  |  |  |  |  |  |  | NI |
| 1019.71 |  |  |  |  |  |  |  |  | NI |
| 1023.52 |  |  |  |  |  |  |  |  | α-linolenoyl-CoA |
| 1025.04 |  |  |  |  |  |  |  |  | linoleoyl-CoA, OPC6-3-ketoacyl-CoA |
| 1029.38 |  |  |  |  |  |  |  |  | Stearoyl-CoA, linoleoyl-CoA |
| 1030.6 |  |  |  |  |  |  |  |  | hexadecanedioyl-CoA |
| 1032.75 |  |  |  |  |  |  |  |  | trans-octadec-2-enoyl-CoA, stearoyl-CoA |
| 1050.7 |  |  |  |  |  |  |  |  | dihomo γ-linolenoyl-CoA, eicosatrienoyl-CoA |
| 1054.7 |  |  |  |  |  |  |  |  | OPC8-3-ketoacyl-CoA, (11Z,14Z)-icosa-11,14-dienoyl-CoA |
| 1056.76 |  |  |  |  |  |  |  |  | trans-arachido-2-enoyl-CoA, phytenoyl-CoA |
| 1060.7 |  |  |  |  |  |  |  |  | pristanoyl-CoA, phytanoyl-CoA |
| 1072.7 |  |  |  |  |  |  |  |  | 3-hydroxy-(11Z)-eicos-11-enoyl-CoA, 3-oxo-arachidoyl-CoA |
| 1074.5 |  |  |  |  |  |  |  |  | (3R)-3-hydroxy-arachidoyl-CoA |
| 1092.67 |  |  |  |  |  |  |  |  | 3-hydroxy-docosapentaenoyl-CoA, dolichol-group |

^1^Cells with a green background indicate that these putative metabolites were significantly increased (*p* = 0.05) in the condition(s) mentioned. Increasingly darker shades of green represent a greater abundance of a given ion.

^2^Cells with a red background indicate that these putative metabolites were significantly decreased (*p* = 0.05) in the condition(s) mentioned. Increasingly darker shades of red represent a greater decrease in abundance of a given ion.

^3^*m*/*z* ions with green or red background indicate that their change in abundance was positively or negatively associated with mycorrhizal colonization efficiency, respectively.

^4^NI = Not identified.
